# The transition to parenthood for Australian heterosexual couples: expectations, experiences and the partner relationship

**DOI:** 10.1186/s12884-018-1985-9

**Published:** 2018-08-22

**Authors:** Damien W. Riggs, Anna Worth, Clare Bartholomaeus

**Affiliations:** 1 College of Education, Psychology and Social Work, Flinders University, GPO Box 2100, Adelaide, 5001 South Australia; 2 School of Psychology, The University of Adelaide, Adelaide, 5005 South Australia; 3 College of Education, Psychology and Social Work, Flinders University, GPO Box 2100, Adelaide, 5001 South Australia

**Keywords:** Heterosexual couples, Parenting expectations, Transition to parenthood

## Abstract

**Background:**

The perinatal period precipitates significant intra- and inter- personal changes. How heterosexual couples understand and account for such changes, however, has received relatively little attention.

**Methods:**

Semi-structured individual interviews were undertaken as part of a longitudinal study on planned first-time parenthood. This article reports on an inductive thematic analysis of a data corpus focused on six interview questions (three from interviews conducted during pregnancy, and three from interviews conducted six months after the birth of the child), derived from interviews with eight individuals (4 women and 4 men) comprising four couples.

**Results:**

In antenatal interviews, the theme of intrapersonal changes differentiated participants by two sub-themes that were then linked to postpartum experiences. Those who ‘prepared for the worst’ reported positive experiences after the arrival of a child, whilst participants who during pregnancy viewed life after the arrival of a child as ‘an unknown’ experienced challenges. Similarly in terms of the theme of interpersonal change, antenatal interviews were linked to postpartum experiences by two sub-themes, such that participants who approached the impending arrival of a child as a team effort reported that the arrival of a child cemented their relationship, whilst participants who expected that the couple relationship would buffer child-related stressors experienced challenges.

**Conclusions:**

Findings highlight the importance of a focus in antenatal education on the psychological effects of new parenthood, and support for the couple relationship during the perinatal period.

## Background

Pregnancy and the transition to parenthood are major adjustment periods within the lives of many adults, with important implications for new parents, couple relationships, and infant development [1]. Research has consistently demonstrated that the perinatal period is often stressful, and can result in both intra and inter personal changes. In terms of negative intrapersonal changes, previous research has identified a relationship between expectations of parenthood and adjustment to the new role [2–6], such that negative prenatal expectations of parenthood are known to predict postnatal difficulties in both mother-infant and marital relationships [4, 6]. At the same time, however, researchers have hypothesised that negative prenatal expectations may be employed by parents as an adaptive cognitive strategy to ‘prepare for the worst’ and thus protect themselves against a negative postnatal experience [7].

In contrast to research on negative expectations, the evidence regarding positive expectations at the intrapersonal level is inconsistent. High prenatal expectations have been associated with improved mood and affective states, adaptive behaviour and marital satisfaction [8]. However, *unmet* expectations – which tend to be unrealistic or overly positive – have been found to influence women’s evaluations of their experience and are associated with difficulties in the transition to parenthood, including postnatal distress amongst mothers [9, 10]. Conversely, research by Delmore-Ko et al. [2] found that expectations incorporating positive aspects of parenting that were present *in conjunction* with expectations of being able to cope with challenges were associated with better adjustment to parenthood. Collectively, these results support the proposition of Churchill and Davis [11] that a ‘Realistic Orientation’ – giving frequent thought to both positive and negative possibilities – better prepares women for the transition to motherhood, and promotes resilience in the face of adversity.

In terms of changes to interpersonal relationships accompanying the transition to parenthood, the literature also suggests that there is a link between prenatal expectations and postnatal outcomes [5, 12, 13]. Previous research has demonstrated that when partners’ expectations are not met, there is an increase in marital conflict, and decline in marital satisfaction – and that this association is more evident in women than men [12]. Delmore-Ko and colleagues [2] found both similarities and differences in women’s and men’s expectations about parenthood, and concluded that discrepancies in partners’ prenatal expectations may put extra stress on partner relationships in the postnatal period. Furthermore, research has demonstrated that any potential decline in relationship satisfaction can be mitigated by greater paternal involvement in infant care [14] and household labour [15], and that the provision of emotional and practical partner support is a strong protective factor against postnatal depression in women [16, 17].

Yet despite the mitigating role that paternal involvement can play in terms of relationship satisfaction amongst heterosexual couples negotiating the transition to parenthood, the transition to parenthood literature has predominantly focused on the role of mothers, with fathers and couples receiving less attention. It has also been noted that much of the literature is dated, and may not accurately reflect the transition to parenthood in a contemporary social context [18]. Additionally, much of the existing literature focuses on the postnatal rather than the antenatal period, leaving this initial period of adjustment relatively understudied, particularly with regard to men undergoing the transition to fatherhood. Furthermore, many studies have asked new parents retrospectively about their expectations and experiences during their pregnancy and the early postnatal period, rather than prospectively.

The current paper seeks to address the aforementioned limitations by undertaking an analysis of in-depth, qualitative longitudinal interview data exploring the antenatal expectations and postnatal experiences of a sample of heterosexual couples living in Australia. The findings presented emphasise experiences that may indicate a positive adjustment to parenthood in both individuals and couples, as well as those experiences that may indicate challenges for individuals and couples, and highlight the importance of support for the couple relationship during the perinatal period.

## Methods

The findings presented in this paper draw on an ongoing qualitative longitudinal study examining the experiences of ten heterosexual couples through their journey to conception, pregnancy, and birth. The study is focused on the desire to have children, decision-making and expectations related to planning for a first child, and subsequent experiences during pregnancy and after the child is born. (For detailed information about the broader study and sample, see [19].) Ethics approval was granted by the Flinders University Social and Behavioural Research Ethics Committee. Potential participants contacted the researchers via email and were emailed an information sheet, consent form, and list of supports. Participants returned their signed consent forms prior to the start of the first interview, by email or in person at the interview.

### Participants

Key selection criteria for inclusion in the study were that people were (1) in a heterosexual relationship (married or de facto), (2) planning for a first child, (3) Australian citizens living in Adelaide, South Australia, and (4) not aware of any significant fertility concerns. Participants were recruited during February–May 2015 by advertising in local media, and on Facebook, Twitter, and a range of internet forums that focus on parenting. These purposive recruitment strategies generated a normative sample of 20 white, middle-class participants, comprising 10 couples.

The current study draws on two rounds of interviews with four couples: Deb and Sam, Cate and Simon, Lucy and Jon, and Mary and Max (all pseudonyms), yielding a total of 16 interviews. All women participating had a bachelor degree or higher, whereas the highest qualification obtained by four of the men was either secondary school or trade certificate. All couples were married and working fulltime in paid employment at the second interview. At the third interview, three of the men were in fulltime paid work and two part-time. Two of the women were in part-time paid employment, two were fulltime carers, and one was studying part-time.

### Procedure

Individual semi-structured interviews occurred (or will occur) over four waves: 1) when the couple was planning a pregnancy via reproductive heterosex, 2) when the couple is six months pregnant, 3) six months after the birth of the child, and 4) 18 months after the birth of the child. The findings reported in the present paper focus on interview data collected at waves 2 and 3. The second interviews conducted focused on the experience of pregnancy, preparation to become a parent, and plans for the birth and early postnatal period. The third interviews focused on experiences at the end of pregnancy, the birth, the first few weeks of parenting, and current experiences now their baby is six months old. Men and women were interviewed separately. Participants selected the method of interview they preferred, with most interviews conducted in person for the first and second rounds, with others conducted via Skype or telephone. All interviews were audio-recorded, with the average length of each interview recording for the five couples discussed in this article being just over 60 min. Recordings were transcribed verbatim by a professional transcription service and participants were allocated pseudonyms by the authors following transcription.

### Analytic approach

Drawing on the literature summarized in the introduction of this paper, an inductive thematic analysis was undertaken, focusing on participants’ accounts of the expectations and reality of new parenthood in terms of change both in intra and interpersonal contexts. Braun and Clarke [20] refer to such an inductive theoretical approach as ‘contextualist’, in that it seeks to understand how people create meaning in their lives, and how this meaning reflects broader social constructs. Given that the focus in this paper is on men’s and women’s experiences, gender as a social construct was a likely contextualising factor shaping how participants viewed the transition to parenthood. For the purposes of the analysis, responses to the following questions from waves two and three of the interviews were examined: “What are your plans for post-birth?”, “What changes do you think will happen in your household?”, and “How do you feel about your relationship with your partner now you are having a child?” (Interview 2) and “Tell me about bringing your baby home from hospital”, “Is having a child like what you expected?”, and “What is your relationship like with your partner now you have a child together?” (Interview 3). For the purposes of the analysis, these two waves were treated as distinct data sets, though connected via the couples contained within each.

Data analysis followed the six phase thematic analysis process outlined by Braun and Clarke [20]: (1) becoming familiar with the data, (2) generating codes, (3) identifying themes, (4) reviewing themes, (5) refining specifics of the themes, and (6) selecting extracts that best illustrate the themes identified. Following repeated readings of the two data sets (wave 2 and 3 interviews), each was coded by the second author. These codes were then used to identify key themes within each data set. In outlining their approach to thematic analysis, Braun and Clarke eschew the need for inter-rater checks of coding, arguing that all analyses of data are subjective, and that two or more researchers cannot be expected to arrive at exactly the same interpretation of the data. Nonetheless, the themes generated by the second author were reviewed and confirmed by the first author.

Given the longitudinal nature of the data, they are presented in three intersecting ways. First, they are presented as themes according to whether they focus on intra or interpersonal change. Second, they are presented by time (i.e., second or third wave interviews). Third, subthemes are presented within each theme differentiated by time. Couples are matched across subthemes, differentiated by time. Due to space constraints, each couple appears under both interview waves of *either* inter or interpersonal change. The couples that appear under each are those that best illustrate the subthemes.

## Results

Participants engaged in detailed discussion regarding their experience of the transition to parenthood. Their accounts referenced two clear overarching themes: *Intrapersonal change*, in which they oriented to issues of individual hopes and fears, and *Interpersonal change*, where participants reflected on the changing nature of their relationship in the context of pregnancy and new parenthood. Within each of these overarching themes, four subthemes were identified (two for each of the waves of data collection included in this paper). These eight subthemes are presented below, with a focus on how couples reported experiencing the transition to parenthood in terms of intra and inter personal change.

### Intrapersonal change

Participants indicated that intrapersonal change captures aspects of the transition to parenthood such as the acquisition of new roles and identities as mothers and fathers, individual expectations, and references to emotions, including notions of ‘coping’ with changes associated with parenthood. In their accounts of intrapersonal change, women discussed their emotions and the challenges of parenting an infant more frequently than men, who accounted for the transition to parenthood in the context of increased responsibility, and broad references to family. Within this overarching theme, subthemes based on second wave interviews were: (1) Preparing for the worst, and (2) Future as unknowable. Subthemes for the third wave interviews were: (1) Better than expected, and (2) Unexpected worries and challenges.

#### Interview 2: Pregnancy

The data collected during pregnancy contained a prevalent ‘future focus’ with regard to intrapersonal change. Participants provided detailed accounts of their thoughts about impending parenthood, often drawing on the parenting experiences of others.

##### Preparing for the worst

Participants located in this subtheme spoke about their expectations for the early postnatal period in terms of needing to prepare for the worst. For Deb and Sam, preparing for the worst involved their own fears, as well as those shared with them by others. Deb, for example, reported the perception that in the weeks following birth most of her regular routines would disappear so as to focus on the baby:

Deb: That’s the period I’m scared of. I just truly imagine that I won’t be doing anything except feeding and trying to get the baby to sleep and me trying to sleep. Remember to go to the toilet and shower every now and again and eat every now and again. So I’m expecting it to be fairly chaotic and completely baby focused at least in those first two weeks and the household and everything else will just fall to disarray.

Drawing on information shared by male friends, Sam too appeared to be preparing for the worst:

Sam: Very difficult and very tough just from what the guys have told me, they don’t shy away from it. It’s the lack of sleep and trying to support the partner through it all. So very tough and a lot of hard work.

Slightly different to Deb, who appeared focused primarily on the baby and if possible remembering to care for herself, Sam appeared focused on supporting Deb through the early weeks following birth.

##### Future as unknowable

This subtheme emphasised the idea of the future as uncertain or indeed unknowable. This appeared to serve the purpose of counteracting worry about the future, however it did not appear to necessarily reflect a realistic orientation (i.e., incorporating both possible positive and negative outcomes), as was true for Lucy and Jon:

Lucy: I think it might be a little bit stressful because we might be like ‘oh, is this normal?’ We won’t know for this first child, though with the second child we might be like yeah, yeah, we know what to do.

Whilst Lucy acknowledges that following the birth of the child things might be ‘a little bit stressful’, her questioning of whether or not things as they unfold will be ‘normal’ doesn’t appear to indicate a clear orientation towards what can reasonably be expected. Jon reported a similar perception:

Jon: First couple of weeks I’ve got no idea and don’t want to plan too much there that’s for sure, I’ve no idea, no idea… It’s going to be weird, the first few months, I mean holding the baby and being like this is my kid, all that kind of stuff.

On the one hand, Jon has potentially positive expectations related to holding the baby, yet on the other hand he does not report an awareness of any potential challenges, instead indicating no intentions to plan ahead.

#### Interview 3: Six months postnatal

In contrast to the future orientation that was evident during pregnancy, data collected six months after birth related to the experience of making sense of the significant intrapersonal changes associated with the postnatal period. Whilst some participants provided accounts of processing, embracing, and accepting change, it was clear that others were still negotiating and coming to terms with the reality of parenthood.

##### Better than expected

Due to their imagined worst case scenarios as outlined in the first subtheme above, Deb and Sam described being pleasantly surprised that the experience of caring for a new baby had met or exceeded their expectations. They both acknowledged that the early postnatal period was challenging, but also indicated their preparedness for this reality, as can be seen in the following quote from Deb:

Deb: I truly think I was expecting it to be the hardest time of my entire life and I don’t necessarily think it was because I think I was quite blessed in that I knew what to expect to some degree. Like I’d seen some people struggle so much with it and I’d seen really difficult breastfeeding experiences, but he slept well and he fed well and I realise that because of those two things it was a lot easier than I was expecting. It wasn’t easy but it was certainly not as challenging as I was expecting. So I think I did feel prepared.

In this quote Deb acknowledges that, compared to others, she has been fortunate that her baby sleeps and feeds well, however she also indicates that being prepared for the worst had served her well. Sam similarly reported that things had gone better than expected:

Sam: you feel surprised that you know this is a bit better than I thought. You know I thought he’d be crying 24/7 but he was rather sleepy. And we had a few visitors come by so that was special as well because it was Deb’s side, like for her parents it’s their first grandchild. So it was a very special moment when her family and that came over and visited.

Despite preparing for the worst in terms of crying and lack of sleep, Sam was pleasantly surprised that things had gone well following the birth of the child. Having family visit during this early period following the birth appeared to further engender a sense of positivity in the face of having prepared for the worst.

##### Unexpected worries and challenges

For Jon and Lucy, it appeared that in seeing the future as unknowable, the challenges of a first child had been greater than might have been the case had their future orientation incorporated awareness of all possible outcomes. For Jon and Lucy, a key challenge related to breastfeeding, as Jon notes:

Interviewer: What about feeding?

Jon: Lucy has been, obviously, the one in control of that. [….] She really had a hard time with that. “What’s wrong with me?” kind of thing. “I want to be the perfect mum.” I think that’s why a lot of mums don’t talk about mastitis, because they feel like they’ve failed or something like that. There were a couple of weeks there where Lucy was quite depressed, quite distraught about it.

Challenges with breastfeeding (and here specifically mastitis) may not be entirely predictable, yet having not even considered the possibility during pregnancy meant that Jon and Lucy were less than prepared, as Lucy notes:

Lucy: I remember feeling so lost, like, “What’s wrong with me?” If I knew that it’s fine, then maybe I wouldn’t have come across as so stressed out with it.

Interviewer: Did you look for information to help you with that or with anything else?

Lucy: I was just asking Jon what should I do? What should we do? He suggested that maybe we can buy a pump and I can express from that breast [....] So, it was good to have Jon there to talk about it. He said, “Look, I’m just going to get you a pump”, and then he just went and did that. That was really good for me.

Not being aware of potential challenges left Lucy feeling stressed, however Jon was able to identify a solution to the challenges.

### Interpersonal change

Interpersonal change captures aspects of the transition to parenthood pertinent to the couple relationship, such as the changing dynamic, relationship intimacy and stress, and descriptions of the source of increased commitment or challenges. Within this overarching theme, subthemes identified during pregnancy were: (1) Pregnancy demonstrates commitment, and (2) Confident that the relationship will mitigate potential stressors. Subthemes identified in the postnatal period were (1) Arrival of baby cements commitment, and (2) Relationship challenges due to stressors.

#### Interview 2: Pregnancy

With regard to interpersonal change, the data collected during pregnancy again contained an overarching ‘future focus’. However, in contrast to their accounts of intrapersonal change during the transition to parenthood – which contained both positive and negative expectations – participants’ descriptions of potential negative interpersonal change appeared to have been reflected on in less depth.

##### Pregnancy demonstrates commitment

Mary and Max experienced pregnancy as a time of increased intimacy and connection in their relationship. For Mary, Max’s actions reaffirmed her views about him as a parenting partner:

Mary: It’s nice and reassuring that he is what I think he is, he is the guy that I thought he was, and we do work like I thought we would. We are the team that I thought we were. So, I think it’s just reaffirmed lots of those things. He’s beautiful. I always saw him as a dad, anyway. It’s really sweet to see him – most nights he goes to sleep with his hand on my belly because he likes feeling the kicks and that kind of stuff.

For Mary, Max’s engagement with the child during the antenatal period signified his commitment to being a father. For Max, it would appear, this commitment was driven by the perception that having a baby constituted a shared project:

Interviewer: How do you feel about your relationship with Mary now you’re having a child?

Max: Really good. Really happy. It has brought us together heaps. There’s just a shared goal or project to do well at, I think, and we’re both pretty excited to see bits and pieces of each other in it.

Whilst different to a degree in nature to Mary’s account, Max nonetheless appeared emotionally driven by the pending arrival of their first child, indicating that he was ‘excited’ about how the baby would reflect them both as parents.

##### Confident that relationship will mitigate potential stressors

For Cate and Simon, there was a sense in which they were both aware that the arrival of a baby would likely bring stressors, but both also indicated that they believed their relationship would help buffer against any stressors. As Cate noted in particular, she was prepared for the worst, but was nonetheless excited:

Cate: Because everyone seems to have those moments where they almost go nuts, I’m thinking, okay, that will happen I just have to prepare myself that will happen, we will have those nights where she will not stop crying or not settle and we’re just so exhausted we’re both about to burst out crying. But we are both extremely excited about being parents and we both feel that the other person will be an amazing parent.

Here Cate reflects that her view of Simon, and vice versa, is that he will be ‘amazing’, potentially indicating a relationship strength that she believes will carry them through any difficult times. Simon specifically noted that they had been ‘through quite a lot of stuff already’ in their relationship, and that this made them ‘suited to stress’:

Simon: I’m not sure how it will affect Cate, like Cate and me as far as our relationship, we’ve gone through quite a lot of stuff already I’d say and so I think we’re fairly well suited to stress and stuff like that. I think we’ll get there it’ll just be a case of trying to function with not a lot of sleep.

Again, Simon acknowledges that he is prepared for the worst, however feels that ‘we’ll get there’, because as a couple he and Cate have managed to navigate challenges in the past.

#### Interview 3: Six months postnatal

At the six-month postnatal interview, participants reported both heightened connection and challenges in their interpersonal relationship. The postnatal data offered richer and more reflective accounts of interpersonal change than those given during pregnancy, reflecting the significant changes to the couple relationship between the two interviews.

##### Arrival of baby cements commitment

For couples such as Mary and Max, the increased intimacy experienced during pregnancy continued to feature in descriptions of their postnatal relationship. For Mary, a description of increased love and relationship satisfaction was associated with the perception of paternal involvement with infant care:

Interviewer: What’s your relationship with Max like now that you have a child together?

Mary: I would say, if anything, better, but it still feels the same to me in most ways. I think because we’ve approached it like as a team, as we always have done for everything else, it…I wouldn’t even say it has bonded us closer because I think we were impossibly closely bonded before. I don’t think there’s ever any closer we could get. And so, yeah, I feel like we’re as close as we’ve ever been and I’m probably more proud of him that I’ve ever been because of the way he interacts with her.

It would appear that Mary’s expectations of Max as a father during the pregnancy were met and surpassed during the period following birth. This would seem to be the case because they functioned as a team, as opposed to a primary caregiver and supportive partner. Max too emphasised an account of them sharing responsibility for the baby when asked about their relationship:

Max: Still - yeah, like really close. Like we pretty much spend like I guess the whole time we’re together with [baby] as well and it doesn’t seem to take anything away from what we had before she was born. So, yeah, still really good. It’s really good to share [baby] growing up with each other and learn about her together.

Here Max is explicit that sharing time together with the baby doesn’t subtract from the happiness they shared as a couple prior to the birth. Indeed, if anything it adds an extra dimension, as he expected it would in his second interview.

##### Relationship challenges due to stressors

For some couples such as Simon and Cate, conflict within the couple relationship was a feature of the early postnatal period. For men such as Simon, accounts of conflict were primarily constructed as a product of fatigue and reduced time alone with their partner:

Interviewer: What’s your relationship like with Cate now you have a child together?

Simon: We, I suppose – I wouldn’t say argue – but when we’re tired and you get a bit snarky at one another, that happens more than it ever did before [….] We certainly have a lot less time for us. That’s one thing that we’ve definitely come across. It is a lot harder to have time for us as a couple, not as individuals.

For Simon, it would appear that some of the past challenges mentioned in his second interview (in terms of getting ‘a bit snarky’) had increased following the arrival of the baby. In part Simon appears to attribute this to the reduction in time they have together as a couple, though for Cate the increased challenges were attributed to feeling that she had to take primary responsibility for the baby:

Cate: The long-term effects of tiredness have meant that I’m a lot more irritable generally. And so, yeah, our relationship has definitely changed and I think we’ve definitely had more conflict. It’s not easy because there’s the feeling of resentment that I have that whenever there’s a problem it will come down to me… And I get this jealousy about the fact that he gets to go to work and leave it all behind, which is stupid because I don’t want to go to work, but there’s a sense of resentment I think that he has the freedom that I don’t have anymore because that’s it, it’s all taken away and I’ll never have that again.

Expecting that the strength of the relationship would mitigate challenges appeared in reality not to be the case for Cate. Feelings of primary responsibility for the baby and that “it’s all taken away and I’ll never have that again” may have exacerbated any conflict that was present prior to the arrival of the baby.

## Discussion

Through an examination of accounts of new parents’ expectations, experiences, and relationships during the transition to first time parenthood, this study has explored important aspects of intrapersonal and interpersonal change during the perinatal period. In terms of intrapersonal change, whilst previous studies have reported that negative prenatal expectations of parenthood predict postnatal difficulties for mothers [4, 6], the participants in our sample who ‘expected the worst’ reported an easier adjustment to the early postnatal period. These findings are consistent with those of Muscat, Thorpe and Obst [7], whose work suggests that some new parents may employ an adaptive cognitive strategy to protect themselves against the possible disappointment associated with a potentially negative parenting experience. Also in contrast to previous research, which has suggested that the ability to embrace the unknown enables women to successfully renegotiate and adjust their expectations according to reality [10, 21], the findings reported in this paper with regard to some couples treating the impending arrival of a baby as ‘unknowable’ (and hence able to be planned for) appeared to lead to challenges in adjusting to the baby when it arrived.

With regard to interpersonal change, participants experienced pregnancy as a time of happiness and connection. Having a child together was central to descriptions of greater commitment, and both men and women expressed positivity with regard to their future relationship. Consistent with previous research [22], and in keeping with their own prenatal expectations, some participants did indeed report experiencing increased connection and relationship satisfaction following the birth of their baby, and this appeared to contribute to a relatively smooth and positive transition to parenthood. By contrast, for participants who reported relationship challenges that pre-existed the pregnancy, it would appear that the relationship was unable to buffer the additional challenges that arose following the birth of a baby. The findings would suggest that key to these differential outcomes was paternal involvement, reflecting previous research demonstrating that decline in relationship satisfaction can be moderated by greater paternal involvement in infant care [14].

## Conclusions

Taken together, the findings reported in this paper suggest that at the intrapersonal level, the development of ‘realistic’ expectations allowed some of couples in the sample to experience the early postnatal period as having met or exceeded their expectations of new parenthood. By contrast, for participants who did not have a clear perception of what might lie ahead, this appeared to be related to poorer outcomes after the birth of the child, though these participants were nonetheless able to identify solutions to challenges as they arose.

At the interpersonal level, the assumption that the couple relationship would mitigate child-related stressors appeared for some participants to be unfounded, whilst for other couples seeing the pregnancy as a joint project that would cement their commitment to one another appeared to be related to more positive outcomes.

These findings suggest potential implications for practice. The majority of antenatal education programs focus on the physical processes of pregnancy, birth and infant care [5], and new mothers report feeling well educated yet psychologically unprepared for parenting [23]. In keeping with recommendations arising from previous research [5, 9, 13, 24], the findings of the present study suggest the need for a greater educational focus on the social and emotional changes associated with parenthood. Specifically, this may include the importance of psychological preparedness (rather than viewing the birth of a child as psychologically unknowable), and recognising that for some people preparing for the worst may be a useful psychological strategy. For couples, the findings suggest that whilst for some the challenges that may come with a new baby cannot be predicted or avoided, presuming that the couple relationship will mitigate challenges may leave some unprepared. This may be especially so if relationship challenges are already present prior to conception and birth. Access to services that encourage couples to explore existing challenges prior to pregnancy and the birth of a child may thus be beneficial.

We acknowledge that when considering the implications and relevance of these findings explored in this paper, there are limitations to the research that should be considered. We particularly note the limitations of the small sample size. However, the depth of the interviews allowed for a detailed exploration of the experiences of participants, and the inclusion of both male and female participants makes this a useful contribution to the literature. It is also possible that the findings may have been influenced by a degree of social desirability. As noted elsewhere [9], the social ideology of motherhood restricts women from speaking negatively about their babies, which may explain the tendency of women in our sample to attribute negative postnatal emotions to the experience of conflict with their partners, rather than to negative thoughts and feelings towards the infant.

In conclusion, the transition to parenthood precipitates an array of intra and inter personal changes that underscore the need for programmes that help facilitate the transition to first time parenthood. Targeting the couple relationship prenatally is an inclusive approach that positions perinatal mental health as a family issue, rather than something specific to women [25–27]. Given the consistent finding of previous research that relationship functioning predicts postnatal distress in both women and men, it is critical that the couple relationship is supported during this time. Such support is vital for the psychological wellbeing of mothers, fathers and infants.
